# A morphological, molecular and life cycle study of the capybara parasite *Hippocrepis hippocrepis* (Trematoda: Notocotylidae)

**DOI:** 10.1371/journal.pone.0221662

**Published:** 2019-08-23

**Authors:** Jordana C. A. Assis, Danimar Lopez-Hernández, Eduardo A. Pulido-Murillo, Alan L. Melo, Hudson A. Pinto

**Affiliations:** Laboratório de Biologia de Trematoda, Department of Parasitology, Institute of Biological Sciences, Universidade Federal de Minas Gerais, Belo Horizonte, Minas Gerais, Brazil; Charles University, CZECH REPUBLIC

## Abstract

*Hippocrepis hippocrepis* is a notocotylid that has been widely reported in capybaras; however, the molluscs that act as intermediate hosts of this parasite remain unknown. Furthermore, there are currently no molecular data available for *H*. *hippocrepis* regarding its phylogenetic relationship with other members of the family Notocotylidae. In the present study, we collected monostome cercariae and adult parasites from the planorbid *Biomphalaria straminea* and in the large intestine of capybaras, respectively, from Belo Horizonte, Minas Gerais, Brazil. We subjected them to morphological and molecular (amplification and sequencing of partial regions of 28S and *cox-1* genes) studies. Adult parasites collected from the capybaras were identified as *H*. *hippocrepis* and the sequences obtained for both molecular markers showed 100% similarity with monostome cercariae found in *B*. *straminea*. The sequences obtained for *H*. *hippocrepis* were compared with data available in public databases; analysis revealed this species differs from other notocotylids with available sequences (1.5–3.8% with respect to 28S and 11.4%–13.8% with respect to *cox-1*). On the phylogenetic analyses, *H*. *hippocrepis* appeared to be a distinct lineage in relation to other notocotylids. Some ecological aspects related to the infection of capybaras with *H*. *hippocrepis* are briefly discussed.

## Introduction

The capybara, *Hydrochoerus hydrochaeris* Linnaeus, 1766, is a semi-aquatic rodent that is widely distributed in South America. Its high reproductive rate and adaptative capacity favor the formation of large social groups in anthropogenically impacted urban environments centers [1–4]. The proximity of these animals to humans is a cause for concern because capybaras, in addition to acting as hosts for several causative agents and vectors of medical and veterinary importance [5–8], also present a variety of helminths, including at least twenty-five species [5, 9–12]. Nevertheless, aspects of the life cycle, pathology, ecology and the zoonotic potential of these parasites remain unknown.

Among the eight species of trematodes already reported in capybaras, *Hippocrepis hippocrepis* (Diesing, 1850) is the species most widely distributed, having been found in Argentina, Bolivia, Brazil, Uruguay and Venezuela (revised by [11]). In some cases, high infection rates (up to 80%) and intensities of infection (up to 19,600 parasites) by this notocotylid have been recorded [13, 14]. Studies other than reports of *H*. *hippocrepis* in its definitive host are non-existent. Therefore, studies aimed at identification of the intermediate host involved in the transmission of *H*. *hippocrepis* remain necessary.

Recently, the use of molecular tools has increased knowledge of trematode biology, including their life cycles [15–19]. Nevertheless, combined morphological and molecular approaches to study species of the family Notocotylidae are scarce [20–25]. In fact, there are no sequences available for species of the genus *Hippocrepis* or other notocotylids from South America. In this sense, molecular tools can clarify aspects yet unknown regarding the life cycle of *Hippocrepis*.

In the present study, cercariae of the monostome type found in molluscs and adult parasites obtained from naturally infected capybaras from Brazil were subjected to morphological and molecular studies. The results allowed us to associate the larval stages and adults morphologically identified as *H*. *hippocrepis*. In addition to presenting the morphological descriptions of the larval stages of this fluke, we used molecular sequences to evaluate the phylogenetic position of *H*. *hippocrepis* in relation to other notocotylids with available molecular data. The new biological information reported here is contextualized with epizootological aspects related to this parasite.

## Materials and methods

### Malacological study and obtaining of larval forms

During long-term malacological studies carried out in two urban waterbodies from Belo Horizonte, Minas Gerais, Brazil, specimens of the planorbid *Biomphalaria straminea* (Dunker, 1848) are frequently found infected with cercariae. Among the various cercarian types morphologically characterized with the aid of classical literature [26–29], we studied a larva belonging to the monostome type. In a first field phase, molluscs were collected at the Pampulha Reservoir (19° 51' 44.77''S and 43° 58' 29.35' 'W) during 55 samplings carried out between 2009 and 2012 (monthly between February 2010 and December 2011). More recently, between January 2017 and March 2018, 15 field expeditions were performed monthly at a lake located at the Administrative Center of the State of Minas Gerais (“Cidade Administrativa Presidente Tancredo Neves”, hereafter referred to as ACSMG) (19° 47' 06.20''S and 43° 57' 11.41''W).

In the laboratory, the molluscs were placed individually in 24-well polystyrene plates containing 2 mL of dechlorinated water per well, subjected to artificial photostimulation for 2 hours and examined under a stereomicroscope to detect the emergence of cercariae. This test was performed on the day of sampling and repeated the next day. Emerged larvae were initially mounted in non-permanent preparations stained with 0.05% Nile blue sulphate or neutral red. A subsample of the emerged cercariae was collected in a glass vial, and killed in hot water (70°C, by about 1 minute). After decantation of the larvae, some of the water was changed by 10% formalin.

The encystment process of the monostome cercariae was followed using a stereomicroscope. Metacercariae were carefully removed from the plate walls using thin metal needles. Aiming to obtain intramolluscan stages, some infected molluscs were compressed between glass plates and dissected under a stereomicroscope. The mature rediae obtained were wet-mounted for morphological study.

### Obtaining adult parasites from capybaras

Young specimens of a male and a female *H*. *hydrochaeris* (35 kg and 15 kg, respectively) were found dead, apparently recently, on the shore of the lake at ACSMG in March and July 2018. The animals were transported to the laboratory where necropsies were performed. Elongated pink and reddish-colored trematodes were found in the middle and end portions of the large intestine and on the fecal surfaces of both animals. Worms were collected using brushes, washed in physiological saline (NaCl 0.85%) and counted. Samples were compressed between glass slides and fixed in 10% formalin.

### Morphological study of developmental stages

Samples of formalin-fixed cercariae and adult parasites were stained with alum acetocarmine, dehydrated in ethanol, diaphanized in beechwood creosote and mounted on permanent slides with Canada balsam. We performed analyses of stained and mounted adults and found that most eggs overlapped in the uteri, hindering morphometric analysis of the polar filaments. Therefore, some fixed parasites were dissected using metal needles to remove eggs from the distal part of the uteri. We also measured eggs obtained from feces processed using the spontaneous sedimentation method.

Morphological and morphometric studies of both permanent and wet-mounted preparations were performed under an Olympus BH2 optical microscope (; Olympus, Tokyo, Japan). Measurements were obtained using a micrometer eyepiece. Photographs were taken with a Leica ICC50 HD digital camera coupled to the microscope Leica DM500 and analyzed in the Leica Application software suite (LAZ EZ), version 2.0 (Leica Microsystems, Wetzlar, Germany). Drawings were made using a camera lucida. Measurements are expressed in micrometers (unless otherwise indicated) and were represented by the mean and standard deviation, with amplitude in parentheses. Samples of the developmental stages studied were deposited in the Collection of Trematodes of the Universidade, Federal de Minas Gerais (UFMG-TRE).

### Molecular study

Samples of monostome cercariae found in *B*. *straminea* from both locations and adult parasites found in *H*. *hydrochaeris* were fixed in 95% ethanol and maintained at -20°C until use. DNA extraction was performed using the Wizard Genomic DNA Purification kit (Promega, Madison, WI), and the dosage of extracted DNA was determined using a microvolume spectrophotometer (NanoDrop ND-1000; Thermo Fisher Scientific, Wilmington, DE). DNA amplifications were performed using the polymerase chain reaction (PCR) in a total volume of 25 μL. In each reaction we used GoTaq Green Master Mix 2× (Promega, Madison, WI), 10 μM of each primer and ~50 ng of DNA. Two molecular markers were used: the nuclear 28S rRNA gene and the mitochondrial cytochrome *c* oxidase subunit 1 gene (*cox-1*). The primers and PCR conditions used were those previously published: Dig12 and 1500R were used for the amplification of ~1200 bp fragment of the 28S [30], whereas the primers JB3 and COI-R-Trema were used to amplify a fragment of ~ 800 bp the *cox-1* [31]. PCR products were initially subjected to agarose gel electrophoresis and then purified with polyethylene glycol 8000 (20%) (Promega, Madison, WI), following the protocol described by Paithankar and Prasad (1991) [32], with slight modifications. Sequencing in both directions was performed using capillary electrophoresis in the ABI3730 sequencer, with the POP7 polymer and the BigDye v3.1 sequencing kit (Applied Biosystems, Inc., Foster City, CA). Consensus sequences were assembled and edited using the program ChromasPro version 2.0.1 (Technelysium Pty Ltd, Tewantin, Australia). Generated contigs and sequences available from GenBank were aligned using Clustal W implemented in MEGA7 [33], and checked by eye for potential errors. The 28S sequences were used for the phylogenetic analyses by Maximum Likelihood (ML) and Bayesian Inference (BI) methods, using the programs MEGA7 and MrBayes v.3.2.6 [34], respectively. The trimmed 28S alignment presented 1027bp and sequences of other 20 species representatives of the superfamily Pronocephaloidea (S1 Table). The best nucleotide substitution model (GTR + G) was determined according to the Bayesian Information Criterion in MEGA7. The paramphistome *Diplodiscus mehrai* Pande, 1937 (KX506857) was used as the outgroup. For ML analysis, the nodal supports were estimated using the bootstrap method with 1000 replications. BI analyses were performed using Markov chain Monte Carlo (MCMC) for 1,000,000 generations and sampling every 100 generations. The first 25% of the trees sampled were discarded as 'burn-in'. The molecular sequences obtained were deposited in GenBank.

### Ethics statement

The molluscs and vertebrate host found dead were collected under the authority by the Brazilian Institute of Environment and Renewable Natural Resources (IBAMA) (SISBIO 52870–1 and 65433–1). The necropsies of the capybaras were approved by the Ethics Committee in Animal Experimentation of the Universidade Federal de Minas Gerais (CEUA-UFMG protocol 254–2018).

## Results

Cercariae of the monostome type were found in 68/16,235 (0.42%) specimens of *B*. *straminea* collected at Pampulha Reservoir. From 55 samplings derived from this reservoir, monostome cercariae were found in 19, with prevalence of infection ranging from 0.07% to 44.89%. At the lake from ACSMG, 229/1,861 (12.3%) specimens of *B*. *straminea* were found harboring the same monostome cercariae, which were present in all 15 malacological collections (prevalence of infection ranged from 3.03% to 35.7%). After crushing the infected molluscs, clusters of rediae were identified at various degrees of development and age in hepatopancreas. Immature and developed cercariae were found free.

During the necropsy of the two specimens of *H*. *hydrochaeris*, we found trematodes in contact with the mucosa of the large intestine, prior to opening the intestine. After the organ was longitudinally sectioned, pink-to-reddish worms were found in contact but not fixed to the intestinal mucosa. Free parasites were found on the surface of the of feces in formation (S1 Fig). Morphological analysis of the parasites allowed the identification of *H*. *hippocrepis*. The molecular study these adults and cercariae found in *B*. *straminea* from both urban waterbodies evaluated revealed conspecificity between these developmental stages, as presented below.

### Descriptions of life cycle stages

#### Cercariae (Fig 1A, 1B, 1C and 1D)

Larvae of the monostome type. Body brownish. Oral sucker subterminal. Pharynx and ventral sucker absent. Presence of three pigmented eyespots; the two lateral ones are larger than the median. Short esophagus, bifurcating near the eyespots. Long intestinal caeca reaching the posterior extremity of the body. Presence of large numbers of brown cystogenic cells. Genital primordium formed by two masses joined by a tubular canal, in the middle region of the larva. Presence of a pair of dorsal-lateral adhesive pockets located in the posterior region of the body. Excretory system with two main collecting tubules that join in the region posterior to the median eyespot (Monostomi group of Rothschild, 1938 [26]), with numerous small excretory granules inside. Cell flame formulae was not determined. Encystment occurred quickly after emergence of the cercariae.

**Fig 1:**
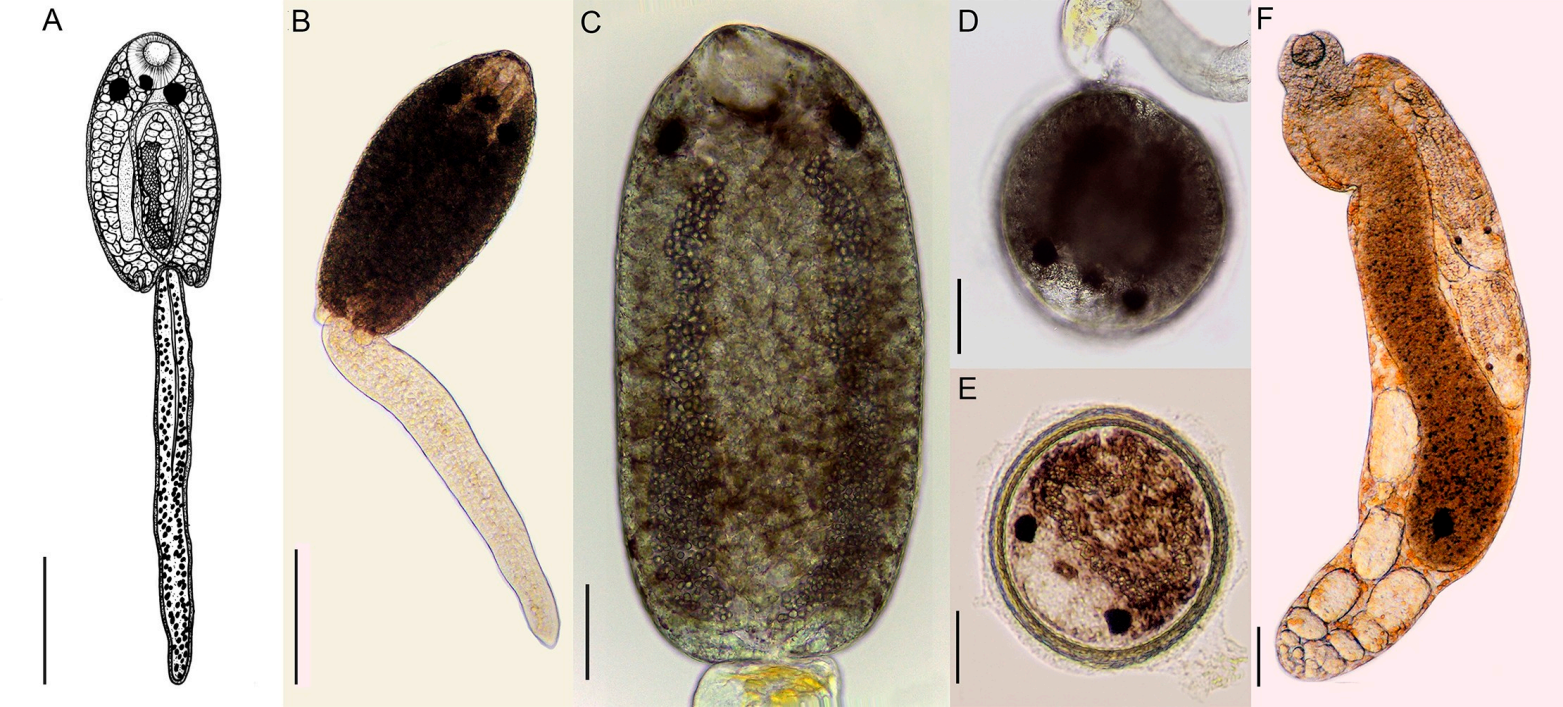
*Hippocrepis hippocrepis*: Larval stages found in *Biomphalaria straminea* from Brazil. Cercaria **(A–C)**, cercaria at initial stage of encystment **(D)**. Encysted metacercaria **(E)**. Mature redia **(F)**. Scale bars: (A-E) 50 μm, (F) 100 μm.

#### Metacercariae (Fig 1E)

Spherical, brown, presenting a thick cystic wall composed of distinct layers. Metacercariae are formed adhered to the walls of the plates as well as on the shells of the molluscs.

#### Rediae (Fig 1F)

Elongated, orange-yellowish, presenting a muscular pharynx and a long and broad caecum, occupying up to 2/3 of the body. Caeca filled by granular material with a brownish spot on the posterior portion, which was verified in all specimens. Presence of about 1–3 developed cercariae and embryonic cercariae (not counted) and several germinal balls. The germinal mass is located at the terminal extremity. The birth pore located anteriorly, at level of pharynx.

#### Adults (Fig 2A, 2B, 2C and 2D)

Elongated, with tegument bearing rows of scale-like spines that are more developed in the anterior region. Oral sucker subterminal, with two lateral papillae. Pharynx and acetabulum absent. Esophagus very short. Cecum long, sinuous, with diverticula, joining posteriorly between the testicles and ending in the posterior portion of the body. Genital pore postbifurcal, median. Cirrus sac well developed, elongated, cirrus long, spinous. Testes opposite, lobed, symmetric, separated by cecal unification, located at the posterior extremity of the body. Ovary median, pre-testicular and intercecal. Mehlis' gland located above the ovary, intercecal. Uterus sinuous, with transverse, intercecal and cecal loops, extending from the anterior third of the body to anterior margin of the Mehlis gland. Vitelline glands bilateral, formed by small follicles, in the posterior third of the body, above the testicles. Transverse vitelline ducts visualized from the last posterior follicles, joining the central region of the Mehlis' gland. In some specimens, few ventral glands were verified in the posterior region. Excretory pore terminal.

**Fig 2:**
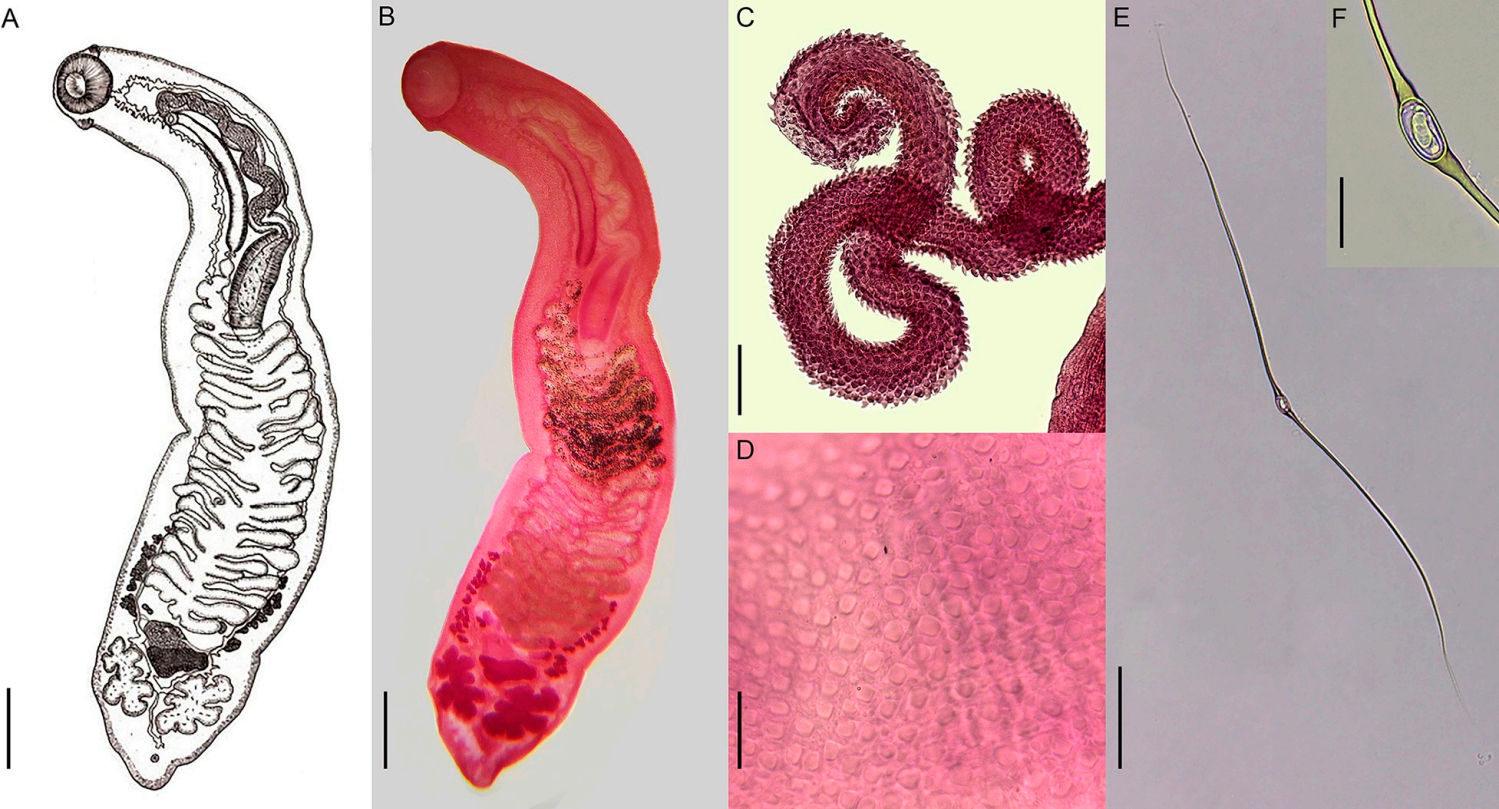
*Hippocrepis hippocrepis*: Adult parasites recovered in *Hydrochoerus hydrochaeris* from Brazil. Whole view of the parasite **(A, B)**. Detail of spined cirrus **(C)**. Detail of tegument bearing scale-like spines **(D)**. Egg of parasites with long polar filament **(E)**. Detail of egg capsule containing the miracidium **(F)**. Scale bars: (A-B) 100 μm, (C) 200 μm, (D) 20 μm, (E) 100 μm, (F) 20 μm.

#### Eggs (Fig 2E and 2F)

Operculated, yellowish, small, with a single very long filament in each pole, progressively becoming sharper. Visualization of the polar filaments was difficult in stained and mounted parasites due the overlapping eggs. Intrauterine eggs obtained after the dissection of formalin-fixed parasites showed tangled filaments, making it difficult to delimit the structure. Eggs obtained from feces present extended polar filaments, facilitating measurement.

Measurements of the larval stages (cercariae, metacercariae and rediae) obtained from naturally infected molluscs are presented in Table 1, which are compared with information available for other monostome cercariae reported in freshwater molluscs from South America. The morphometric data obtained for *H*. *hippocrepis* from capybaras and measures available for *Hippocrepis* spp. in South America are shown in Table 2.

**Table 1:** Morphometric data of cercariae, metacercariae and rediae of *Hippocrepis hippocrepis* found in *Biomphalaria straminea* from Brazil and measures of larval notocotylids reported in *Biomphalaria* spp. from South America by different authors. Data are presented in micrometer (unless otherwise indicated) and are given by mean followed by the standard deviation and range between parentheses. Abbreviations: L: length; W: width.

Species		*Hippocrepis hippocrepis*	*H*. *fuelleborni*	*Cercaria guaibensis* 4	Notocotylidae	*Notocotylus biomphalariae*
Reference		Present study	Ostrowski	Veitenheimer-	Hamman *et al*. (1993) [50]	Flores and Brugni (2005) [49]
			de Núñez (1976) [46]	Mendes *et al*. (1995) [51]		
Host		*Biomphalaria straminea*	*B*. *peregrina*	*B*. *tenagophila*	*Drepanotrema lucidum*	*B*. *peregrina*
Locality		Brazil	Argentina	Brazil	Argentina	Argentina
**Cercariae (*n* = 20)**						
Body	L	291 ± 16 (273–314)	233–350	140–315	348 ± 32 (315–394)	369 (346–394)
	W	132 ± 10 (109–150)	116	99–138	150± 17 (134–171)	191 (154–221)
Oral sucker	L	41 ± 1 (37–43)	40–45	34–58	48± 7 (36–58)	42 (36–53)
	W	41 ± 2 (38–45)	32–45	–	–	41 (34–50)
Lateral eyespots	L	19 ± 2 (15–23)	1 8	14–16	–	–
	W	19 ± 3 (15–25)	–	–	–	–
Median eyespot	L	13 ± 2 (10–15)	–	8	–	–
	W	12 ± 2 (10–15)	–	–	–	–
Tail	L	334 ± 53 (205–403)	360–371	190–410	416 ±52 (360–495)	702 (634–797)
	W	53 ± 6 (41–68)	31	28–48	66 ±11 (54–81)	62 (55–77)
**Metacercariae (*n* = 50)**	L	146 ± 6 (137–163)	156–175	140–196	–	152 (146–158)
	W	143 ± 6 (128–154)	148–168	–	–	–
Cystic wall	L	15 ± 3 (9–21)	13	14–22	–	14 (10–17)
**Rediae (*n* = 30)**						
Body	L	1358 ± 199 (997–1891)	1272	–	–	1013 (643–1248)
	W	277 ± 39 (189–361)	310	–	–	218 (173–307)
Pharynx	L	54 ± 5 (47–67)	59	–	–	68 (58–86)
	W	58 ± 6 (47–75)	64	–	–	73 (58–96)
Caecum	L	996 ± 219 (602–1547)	–	–	–	600 (288–893)
	W	164 ± 36 (102–239)	–	–	–	–

**Table 2:** Morphometric data obtained *Hippocrepis hippocrepis* found in capybaras from Belo Horizonte, Minas Gerais Brazil and data reported to *Hippocrepis* spp. from South America by different authors. Data are presented in micrometer (unless otherwise indicated) and are given by mean followed by the standard deviation and range between parentheses. Abbreviations: L: length; W: width.

Species		*Hippocrepis hippocrepis*			*H*. *fuelleborni*	*H*. *myocastoris*
Reference		Present study	Kohn and Pereira (1970) [38]	Sutton et al. (1997) [39]	Travassos and Vogelsang, (1930) [43]; Kohn and Pereira (1970) [38]	Flores et al. (2007) [45]
Host		*Hydrochoerus hydrochaeris*	*H*. *hydrochaeris*	*H*. *hydrochaeris*	*Myocastor coypus*	*M*. *coypus*
Locality		Brazil	Brazil	Argentina	Uruguay	Argentina
Body	L	7.9 ± 1.4 (6.0–10) mm	6–17.5 mm	8.8 (2.93–11.9) mm	6–8 mm	4.6 ± 0.85 (2.8–6.4) mm
	W	1.65 ± 275.4 (1.01–2.23) mm	1.3–3.35 mm	1.8 (0.71–2.5) mm	1–1.5 mm	1.2 ± 0.26 (0.79–1.6) mm
Oral sucker	L	593 ± 63 (453–714)	360–960	670 (320–890)	370–410	400 ± 60 (250–520)
	W	646 ± 115 (453–907)	480–1080	780 (280–850)	350–370	450± 60 (310–540)
Cirrus sac	L	2.6 ± 0.5 (1.8–3.4) mm	1.6–5.73 mm	3.46 (1.04–4.81) mm	1.71–1.97 mm	1.29 ± 0.28 (0.76–2.17) mm
Right testes	L	737 ± 105 (485–1052)	680–1600	750 (300–980)	710–850	620± 90 (400–810)
	W	549 ± 98 (329–729)	480–1060	580 (240–860)	620–710	420± 70 (280–520)
Left testes	L	740 ± 145 (379–961)	680–1380	740 (270–910)	710–850	610 ± 110 (360–860)
	W	564 ± 115 (363–843)	480–860	550 (240–760)	620–710	400 ± 70 (280–520)
Ovary	L	280 ± 59 (186–400)	260–680	330 (90–430)	480–520	210 ± 40 (130–340)
	W	634 ± 90 (479–843)	480–900	480 (160–630)	310–420	370 ± 50 (250–450)
Mehlis’ gland	L	230 ± 45 (143–336)	190–540	400 (310–460)	–	–
	W	448 ± 85 (300–593)	360–700	540 (430–600)	–	–
Eggs	L	22 ± 2 (19–27)	18–23	20 (16–25)	27–29	26 ± 1 (24–26)
	W	12 ± 1.4 (11–16)	9–14	10 (9–10)	13	13 ± 1 (12–14)
Egg filaments	L	389 ± 36 (314–454)	141–149	-	Absent	54 ± 16 (19–72)

### Molecular study

**Fig 3:**
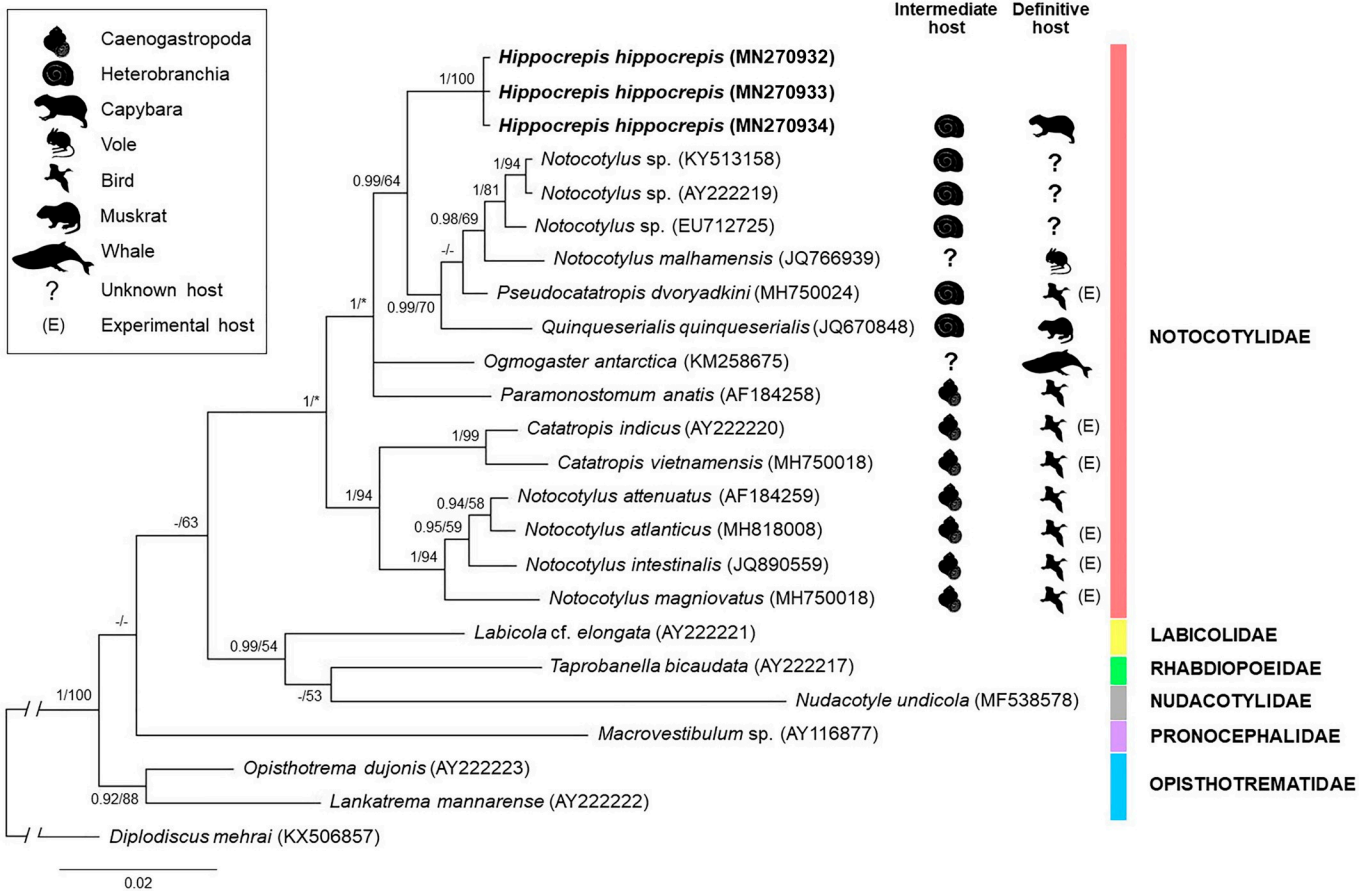
Phylogenetic relationship between *Hippocrepis hippocrepis* (in bold) and other species of the superfamily Pronocephaloidea, as inferred from sequences of 28S rDNA analyzed by Bayesian Inference (BI) and Maximum Likelihood (ML) methods. Nodal support is indicated as BI/ML; values < 0.90 (BI) and < 50 (ML) are indicated by a dash. Asterisks indicate clades that were not present in tree obtained by ML.

Partial sequences of 28S (1172 bp) and *cox-1* (777 bp) were obtained for *H*. *hippocrepis* recovered from capybaras (one sequence for each molecular markers) and for monostome cercariae obtained from *B*. *straminea* (one sequences for each molecular markers and sampling area). Analyses of these sequences showed 100% similarity between adult parasites and cercariae, revealing conspecificity. Moreover, these sequences differed from sequences available in GenBank, as verified by BLAST searches (similarity ≤ 98% and ≤ 90% for 28S and *cox-1*, respectively). Considering the 28S sequences, *H*. *hippocrepis* differed 1.6%–3.8% from 13 species belonging to four genera (*Notocotylus*, *Pseudocatatropis*, *Catatropis* and *Quinqueserialis*) from the family Notocotylidae with sequences available for comparison. The phylogenetic analysis based on 28S sequences performed by ML and BI (Fig 3) revealed similar topology, except by the disposition of *Ogmogaster antarctica* Johnston, 1931 and *Paramonostomum anatis* Garkawi, 1965 and by the grouping between *Pseudocatatropis dvoryadkini* Izrailskaia, Besprozvannykh, Tatonova, Nguyen & Ngo, 2019 and *Quinqueserialis quinqueserialis* (Barker and Laughlin, 1911). All notocotylids included in the analysis formed a well-supported clade. *Hippocrepis hippocrepis* is presented as a distinct lineage in relation to other species evaluated. It presents a sister relationship with a clade conformed by *Notocotylus malhamensis* Boyce, Hide, Craig, Harris, Reynolds, Pickles & Rogan, 2012 + *Q*. *quinquiserialis* + three *Notocotylus* spp. found in freshwater molluscs + *P*. *dvoryadkini*. Interestingly, except for *N*. *malhamensis*, whose intermediate hosts remain unknown, all other species from this clade are transmitted by heterobranch molluscs. In addition, we observed a well-supported clade conformed by species of notocotylids recovered from naturally [*Notocotylus attenuatus* (Rudolphi, 1809)] or experimentally (*Catatropis indicus* Srivastava, 1935, *Catatropis vietnamensis* Izrailskaia, Besprozvannykh, Tatonova, Nguyen & Ngo, *Notocotylus intestinalis* Tubangui, 1932, *Notocotylus atlanticus* Stunkard, 1966, *Notocotylus magniovatus* Yamaguti, 1934) infected birds. The intermediate hosts known for the most species in this clade are caenogastropods, except *N*. *attenuatus*.

In the case of sequences *cox-1*, we found scarcity of data for notocotylids, with only three species identified up to the specific level. Our isolate of *H*. *hippocrepis* differs from *Tristriata anatis* Belopolskaia, 1953, *Ogmocotyle sikae* (Yamaguti, 1933) and *O*. *antarctica* in 11.8%, 17.4% and 11.4%, respectively. Moreover, significant differences (13.4%–13.8%) were also verified in relation to *cox-1* sequences obtained from cercariae of unidentified species of notocotylids obtained from *Austrolittorina antipodum* (Philippi, 1847) and *Austrolittorina unifasciata* (Gray, 1826) from New Zealand and Australia, respectively.

### Taxonomic summary

*Hippocrepis hippocrepis* (Diesing, 1850)

Definitive host: *Hydrochoerus hydrochaeris* Linnaeus, 1766 (Rodentia: Caviidae).

Site of infection: middle and final portion of the large intestine and initial portion of the rectum.

Intensity of infection: about 400 and 1,300 worms.

Locality: Belo Horizonte, Minas Gerais, Brazil

Intermediate host: *Biomphalaria straminea* (Dunker, 1848) (Mollusca: Planorbidae).

Voucher specimens: UFMG-TRE 115–117.

Sequences deposited at GenBank: accession numbers MN270932-MN270934 (28S) and MN268535-MN268537 (*cox-1*).

## Discussion

The species of the genus *Hippocrepis* Travassos, 1922 are trematodes belonging to the family Notocotylidae [35], with three species currently described parasitizing semi-aquatic rodents in South America [11]. The species with the largest number of records is *H*. *hippocrepis*, the type species of the genus described from *H*. *hydrochaeris* from Brazil [36] and later reported in these hystricomorphs by several authors [13, 14, 37–42]. The two other species, *Hippocrepis fuelleborni* Travassos and Vogelsang, 1930 and *Hippocrepis myocastoris* Babero, Cabello and Kinoed, 1979, were described infecting the coypu, *Myocastor coypus* (Molina, 1782) in the Southern region of Brazil, Argentina and Uruguay [37, 43–46]. More recently, *H*. *fuelleborni* was reported in *H*. *hydrochaeris* from the high Paraná River floodplain, and cases of coinfection with *H*. *hippocrepis* were described for the first time [47].

The parasites obtained from capybaras in the present study have morphology and measures similar to those described for *H*. *hippocrepis* by different authors [35–39]. They differ from *H*. *fuelleborni* according to a set of morphological traits, including the symmetrical position of testes (oblique in *H*. *fuelleborni*), absence or scarcity of ventral glands (numerous and distributed from the cirrus pouch to the posterior extremity of the body in *H*. *fuelleborni*), disposition of vitelline glands, which are restricted to the posterior third of the body (occupying half of the body in *H*. *fuelleborni*), and eggs with two polar filaments, one at each pole (eggs without filaments in *H*. *fuelleborni*) [36, 37, 43, 44]. Regarding *H*. *myocastoris*, although it also presents symmetrical testes and does not have ventral glands, as verified in *H*. *hippocrepis*, the extension of vitelline glands is greater and the eggs possess two pairs of filaments in one of the polar regions [45].

Important aspects observed in our study are the considerable differences verified in the measurements of the polar filaments between intrauterine eggs and those obtained from fecal material. The filaments of eggs obtained from feces were almost twice the size of those described for *H*. *hippocrepis* by Kohn and Pereira (1970) [38] (314–454 μm *vs* 141–149 μm). We consider this difference to be a preparation artifact, probably resulting from the difficulty in visualizing the progression of these filaments in intrauterine eggs, or because of the analyses of eggs with filaments not being completely developed. According to ultrastructural studies performed with another notocotylid (*Q*. *quinqueserialis*), the polar filaments are formed as the eggs pass through the uterus, probably being secreted by the walls of this structure [48]. In fact, the presence of eggs with filaments at various stages of development was reported in *H*. *myocastoris* [45]. In the present study, except when eggs were present in the metraterm (verified in few specimens) it was difficult to determine with accuracy the size of the polar filament in eggs from mounted specimens, given that these filaments are clustered. Despite the fact that the dissection of adult parasites to obtain eggs from the distal portion of uteri allowed us to obtain eggs isolates, their filaments were tangled, making the process of measurement somewhat laborious. On the other hand, eggs obtained from feces presented extended filaments, facilitating measurement. Therefore, we suggest that, when possible, morphometric analysis of the eggs of species of Pronocephaloidea should be performed from fecal material. Furthermore, we believe that the presence and size of the filaments should not be considered an isolated criterion for the identification of species of *Hippocrepis*.

To the best of our knowledge, this report presents the first molecular sequences for a species of *Hippocrepis*, which were used for a phylogenetic analysis. Although the number of representatives with available sequences was very small, the tree obtained from the 28S sequences suggests that the group of definitive hosts (mammals or birds) does not represent a phylogenetic signal. In fact, *H*. *hippocrepis* and other two notocotylids of rodents (*N*. *malhamensis* and *Q*. *quinqueserialis*) are presented in a clade together with a bird parasite (*Pseudocatatropis dvoryadkini* Izrailskaia, Besprozvannykh, Tatonova, Nguyen & Ngo, 2019). On the other hand, although not part of the main focus of this work, we emphasize that the topology of the phylogenetic trees obtained suggest that the genus *Notocotylus* is paraphyletic, suggesting the need for additional studies, especially to obtain molecular data for the type species of the genus, *Notocotylus triserialis* Diesing, 1839. The use of various groups of intermediate hosts (Heterobranchia or Caenogastropoda) by the species of *Notocotylus* grouped in different clades or in a same clade (in the case of *N*. *attenuatus*) is curious and requires further analysis. In relation to *cox-1* sequences, *H*. *hippocrepis* differs significantly (11.4%–17.4%) from the few sequences of the members of the superfamily Pronocephaloidea with available sequences, supporting the notion that the genus *Hippocrepis* is distinct from other genera with available sequences. The future acquisition of *cox-1* sequences for members of the family Notocotylidae will allow more robust phylogenetic analyzes resulting in new insights into the taxonomic classification of this group of trematodes.

*Hippocrepis fuelleborni* was the only species of the genus *Hippocrepis* with a previously elucidated life cycle. During studies performed by Ostrowski de Núñez (1976) [46] in Argentina, monostome cercariae found in *Biomphalaria peregrina* (d'Orbigny, 1835) were used to perform experimental infection of rats, resulting in obtaining immature adults identified as *H*. *fuelleborni*. Reviewing the descriptions and illustrations presented by this author, we verified that the testes of these immature parasites were opposite, different from the oblique disposition described for the testes of *H*. *fuelleborni* [37, 38, 43, 44]. It is possible that this difference is due to the analysis of undeveloped parasites that measured on average 2 mm, but that had ventral and vitelline glands similar to those described for *H*. *fuelleborni*.

Thirteen species of the family Notocotylidae are reported from vertebrate hosts in South America [12]. Nevertheless, life cycle studies involving larval stages of these parasites found in molluscs are scarce. The measurements of the cercariae, metacercariae and rediae described here for *H*. *hippocrepis* are similar to those described for *H*. *fuelleborni* by Ostrowski de Núñez (1976) [46]. Considering other cercariae reported in planorbids from South America, the cercariae of *Notocotylus biomphalariae* Flores and Brugni, 2005 found in *B*. *peregrina* and described from experimentally obtained parasites in Argentina [49] are much larger than the cercariae of *H*. *hippocrepis* here reported. An unidentified species of Notocotylidae reported in *Drepanotrema lucidum* (Pfeiffer, 1839) also in Argentina [50], and a larva described as *Cercaria guaibensis* in Southern Brazil [51], also are significantly larger than cercariae here described.

Attempts to infect mice, ducks and chickens with metacercariae now identified as *H*. *hippocrepis* have been performed; however, necropsies performed from 7 days-post infection did not result in obtaining adults for taxonomic identification [52]. In the present study, the association between larval stages found in *B*. *straminea* and adults of *H*. *hippocrepis* recovered from capybaras was possible using molecular analyses. The 28S and *cox-1* sequences obtained for cercariae and adults were identical, revealing conspecificity between these developmental stages. The use of molecular tools becomes promising for the link between developmental stages of notocotylid trematodes, as verified for some species in Europe and Asia [23–25]. However, the molecular approach depends on the existence of available sequences for the respective adult parasites, which are non-existent for most species of Notocotylidae, making specific or generic identification by the molecular approach impossible, as shown by some authors [21, 53, 54]. In fact, the sequences obtained in the present study for *H*. *hippocrepis*, together with *O*. *antarctica*, represent the only sequences available for species of the family Notocotylidae from South America. In this sense, new combined morphological and molecular studies of larval and adult parasites obtained from the final hosts will contribute to the specific identification of new interactions between molluscs and notocotylids.

Considering the biology of the hosts involved in the life cycle of *H*. *hippocrepis* here elucidated, some factors that probably favor parasite transmission can now be discussed. In relation to the definitive hosts, capybaras foraging in the late hours of the morning and in the late afternoon when occurs the ingestion of vegetation next to the shores [1–4], which can present metacercariae of the parasite. On the other hand, *B*. *straminea*, the first intermediate host here reported for *H*. *hippocrepis*, is also widely distributed in South America [55], sharing the same aquatic habitat with *H*. *hydrochaeris*. Feces of this vertebrate can be used as food by the snails, affording the opportunity for parasite eggs to be ingested. Although additional studies are necessary, it appears that there is a host specificity of *H*. *hippocrepis* for *B*. *straminea*, given that two other *Biomphalaria* species [*Biomphalaria glabrata* (Say, 1818) and *Biomphalaria tenagophila* (d'Orbigny, 1835)] occurring in one of the evaluated waterbodies (Pampulha Reservoir) have never been found harboring cercariae of this parasite [52].

Previous reports demonstrated that the prevalences and intensities of infection with *H*. *hippocrepis* from capybaras are quite variable, with reports of prevalence of infection of 20%–80% and intensity of infection from 1–19,600 specimens [13, 14, 39–42]. This variability in these ecological parameters can be related to several factors, including the size of the host population involved in the parasite's biological cycle (capybaras and molluscs) and the type of environment in which the life cycle of the parasite is maintained. In fact, the population density of capybaras in human-populated areas may be significantly higher than that of groups living in natural environments [56]. In fact, the two lagoons evaluated in the present study are man-made urban bodies of water, surrounded by areas for grazing including forage plants on the shores. In these sites, *B*. *straminea* occur with high percentage of infection (up to 44.89%), with larvae identified as *H*. *hippocrepis* verified in various malacological collections. The dead capybaras carried high worm burdens (400 and 1,300 parasites), possibly the result of greater transmission of the parasite in densely urbanized areas. To be sure, the concentration and restriction of infected capybaras in urban waterbodies containing susceptible intermediate hosts favor the transmission of the parasite. The macroscopic alteration in the mucosa of the large intestine caused by parasitism by *H*. *hippocrepis* apparently was not related to damage of the host, probably because the parasite was feeding on the luminal contents. This low pathogenic potential of the parasite can also favor the occurrence of high worm burdens. New in-depth studies related to the interaction between *H*. *hippocrepis* and capybaras are important to elucidate ecological aspects related to the life cycle of this notocotylid.

## Conclusion

This morphological and molecular study of adult parasites found in capybara and cercariae emerged from molluscs made it possible to elucidate the life cycle of *H*. *hippocrepis*. The involvement of *B*. *straminea* as a natural intermediate host of the parasite was described. In addition to the morphological characterization of larval stages of the parasite, for the first time, we presented the molecular sequences and the evaluation of the phylogenetic position of *H*. *hippocrepis* in relation to other members of the family Notocotylidae.

## Supporting information

S1 Fig Macroscopic aspects verified during the necropsy of *Hydrochaeris hydrochaeris* from Brazil. Trematodes identified as *Hippocrepis hippocrepis* were found in contact with the mucosa of the large intestine, visualized prior to the section of the organ **(A, B)**. After the organ was longitudinally sectioned, pink-to-reddish worms were found in contact but not fixed to the intestinal mucosa **(C)** or free on the surface of the of feces in formation **(D)**. Scale bars: 1 cm.
(DOCX)

S1 Table Information on sequences of the region 28S of representative species of the superfamily Pronocephaloidea used for considered in the phylogenetic analysis. (DOCX)
