# Supplementary material for: A morphological, molecular and life cycle study of the capybara parasite Hippocrepis hippocrepis (Trematoda: Notocotylidae)
Source: PLoS One. 2019 Aug 23;14(8):e0221662. doi: 10.1371/journal.pone.0221662 (PMC6707557; doi:10.1371/journal.pone.0221662)
Supplement: S1 Fig — Trematodes identified as Hippocrepis hippocrepis were found in contact with the mucosa of the large intestine, visualized prior to the section of the organ (A, B). After the organ was longitudinally sectioned, pink-to-reddish worms were found in contact but not fixed to the intestinal mucosa (C) or free on the surface of the of feces in formation (D). Scale bars: 1 cm. (DOCX) [file pone.0221662.s001.docx]

**
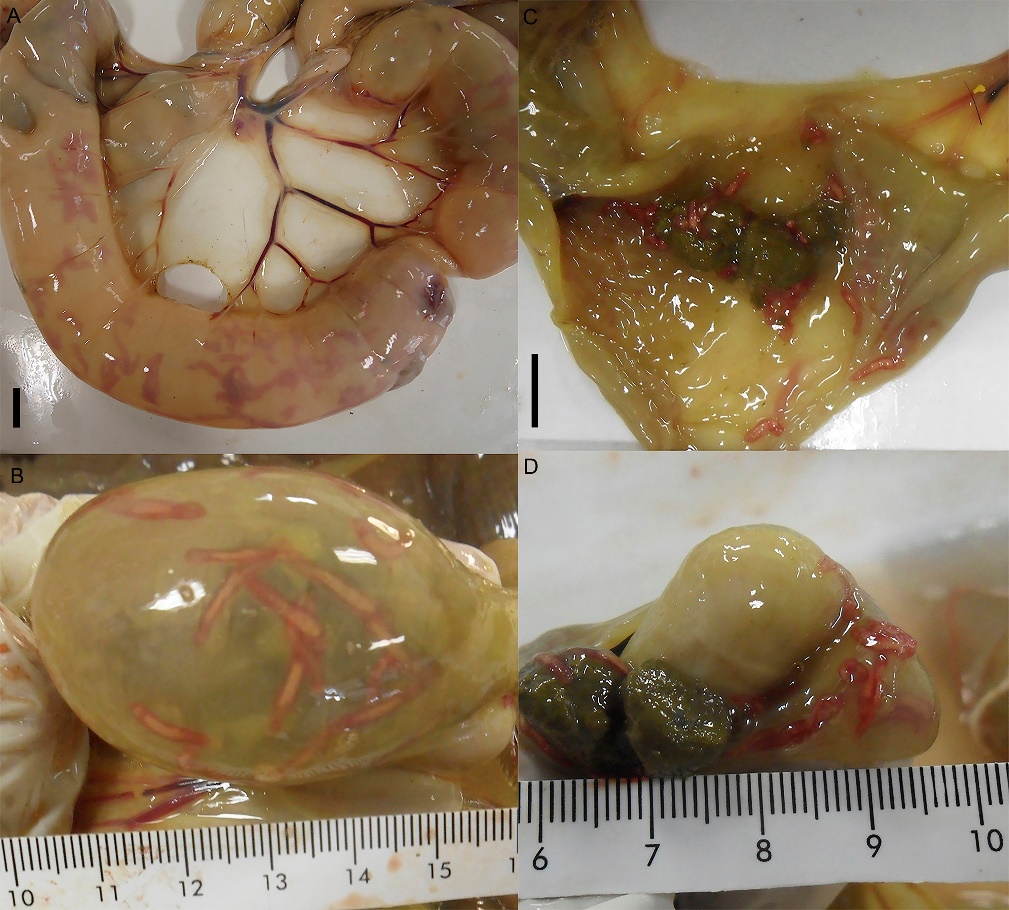
S1 Fig.** Macroscopic aspects verified during the necropsy of *Hydrochaeris hydrochaeris* from Brazil. Trematodes identified as *Hippocrepis hippocrepis* were found in contact with the mucosa of the large intestine, visualized prior to the section of the organ **(A, B)**. After the organ was longitudinally sectioned, pink-to-reddish worms were found in contact but not fixed to the intestinal mucosa **(C)** or free on the surface of the of feces in formation **(D).** Scale bars: 1 cm.
