# Supplementary material for: A morphological, molecular and life cycle study of the capybara parasite Hippocrepis hippocrepis (Trematoda: Notocotylidae)
Source: PLoS One. 2019 Aug 23;14(8):e0221662. doi: 10.1371/journal.pone.0221662 (PMC6707557; doi:10.1371/journal.pone.0221662)
Supplement: S1 Table — (DOCX) [file pone.0221662.s002.docx]

**S1 Table**. Information on sequences of the region 28S of representative species of the superfamily Pronocephaloidea used for considered in the phylogenetic analysis.

| Species | Accession number | Stage | Host | Locality | References |
| --- | --- | --- | --- | --- | --- |
| *Catatropis indicus* | AY222220 | Adult | *Cairina moschata*  (Experimental) | Australia | Olson et al. (2003) |
| *Catatropis vietnamensis* | MH750018 | Adult | *Anas platyrhynchos*  (Experimental) | Vietnam | Izrailskaia et al. (2019) |
| *Labicola* cf. *elongate* | AY222221 | Adult | *Dugong dugon* | Australia | Olson et al. (2003) |
| *Lankatrema mannarense* | AY222222 | Adult | *Dugong dugon* | Australia | Olson et al. (2003) |
| *Macrovestibulum* sp. | AY116877 | Adult | *Trachemys scripta* | USA | Olson et al. (2003) |
| *Notocotylus* sp. | AY222219 | Sporocyst | *Stagnicola palustris* | United Kingdom | Olson et al. (2003) |
| *Notocotylus* sp. | EU712725 | Cercaria | *Physa gyrina* | USA | Hanelt (2009) |
| *Notocotylus* sp. | KY513158 | Cercaria | *Radix balthica* | Norway | Soldanova et al. (2017) |
| *Notocotylus atlanticus* | MH818008 | Adult | *Anas platyrhynchos*  (Experimental) | Russia | Gonchar et al. (2019) |
| *Notocotylus attenuatus* | AF184259 | Adult | *Aythya ferina* | Ukraine | Tkach et al. (2001) |
| *Notocotylus intestinalis* | JQ890559 | Adult | *Gallus gallus domesticus* (Experimental) | Vietnam | Besprozvannykh et al. (2013) |
| *Notocotylus magniovatus* | MH750018 | Adult | *Gallus gallus domesticus*  (Experimental) | Russia | Izrailskaia et al. (2019) |
| *Notocotylus malhamensis* | JQ766939 | Adult | *Myodes glareolus*  *Microtus agrestis* | United Kingdom | Boyce et al. (2012) |
| *Nudacotyle undicola* | MF538578 | Egg | *Trichechus manatus manatus* | Colombia | Velez et al. (2018) |
| *Ogmogaster antarctica* | KM258675 | Adult | *Balaenoptera borealis* | Argentina | Fraija-Fernadez et al.  (2015) |
| *Opisthotrema dujonis* | AY222223 | Adult | *Dugong dugon* | Australia | Olson et al. (2003) |
| *Paramonostomum anatis* | AF184258 | Adult | *Tringa erythropus* | Ukraine | Tkach et al. (2001) |
| *Pseudocatatropis dvoryadkini* | MH750024 | Adult | *Anas platyrhyncha* (Experimental) | Russia | Izrailskaia et al. (2019) |
| *Quinqueserialis quinqueserialis* | JQ670848 | Adult | *Ondrata zibethicus* | USA | Detwiler et al. (2012) |
| *Taprobanella bicaudata* | AY222217 | Adult | *Dugong dugon* | Australia | Olson et al. (2003) |

**References**

Besprozvannykj VV, Ngo HD, Ha NV, Hung NM, Rozhkovan KV, Ermolenko AV. Descriptions of digenean parasites from three snail species, *Bithynia fuchsiana* (Morelet), *Parafossarulus striatulus* Benson and *Melanoides tuberculata* Müller, in North Vietnam. Helminthologia. 2013;50:190-204.

Boyce K, Hide G, Craig PS, Harris PD, Reynolds C, Pickles A, Rogan MT. Identification of a new species of digenean *Notocotylus malhamensis* n. sp. (Digenea: Notocotylidae) from the bank value (*Myodes glareolus*) and the field vole (*Microtus agrestis*). Parasitology. 2012;139:1630-1639.

Detwiler JT, Zajac AM, Minchella DJ, Belden LK. Revealing cryptic parasite diversity in a definitive host: echinostomes in muskrats. J Parasitol. 2012;98:1148-1155.

Fraija-Fernandez N, Olson PD, Crespo EA, Raga JA, Aznar FJ, Fernandez M**.** Independent host switching events by digenea parasites of cetaceans inferred from ribossomal DNA. Int J Parasitol. 2015;45:167-173.

Gonchar A, Jouet D, Skirnisson K, Krupenko D, Galaktionov KV. Transatlantic discovery of *Notocotylus atlanticus* (Digenea: Notocotylidae) based on life cycle data. Par Res 2019;118:1445-1456.

Hanelt B. Hyperparasitism by *Paragordius varius* (Nematomorpha: Gordiida) larva of Monostome redia (Trematoda: Digenea). J Parasitol. 2009;95:242-243.

Izrailskaia AV, Besprozvannukh VV, Tatonova YV, Nguyen HM, Ngo HD. Developmental stages of *Notocotylus magniovatus* Yamaguti, 1934, *Catatropis vietnamensis* n. sp., *Pseudocatatropis dvoryadkini* n. sp., and phylogenetic relationships of Notocotylidae Lühe, 1909. Par Res. 2019;118:469-481.

Olson PD, Cribb TH, Tkach VV, Bray RA, Littlewood DT. Phylogeny and classification of the Digenea (Platyhelminthes: Trematoda). Int J Parasitol. 2003;33:733-755.

Soldanova M, Georgieva S, Rohacova J, Knudsen R, Kuhn JA, Henriksen EH, Siwertsson, A, Shaw JC, Kuris AM, Amundsen PA, Scholz T, Lafferty KD, Kostadinova A. Molecular analyses reveal high species diversity of trematodes in a sub-Artic lake. Int J Parasitol. 2017;47:327-345.

Tkach VV, Pawlowski J, Mariaux J, Swiderski Z. Molecular phylogeny of the suborder Plagiorchiata and its position in the system of Digenea. Int J Parasitol. 2001;30:83-93.

Velez J, Hirzmann J, Lange MK, Chaparro-Gutierrez JJ, Taubert A, Hermosilla C. Occurrence of endoparasites in wild Antillean manatees (*Trichechus manatus manatus*) in Colombia. Int J Parasitol-Par. 2018;7:54-57.
